# Prognostic implication of serum glycated albumin for patients with non-ST-segment elevation acute coronary syndrome undergoing percutaneous coronary intervention

**DOI:** 10.1186/s12933-022-01446-3

**Published:** 2022-01-19

**Authors:** Chi Liu, Qi Zhao, Xiaoteng Ma, Yujing Cheng, Yan Sun, Dai Zhang, Xiaoli Liu, Yujie Zhou

**Affiliations:** Department of Cardiology, Beijing Anzhen Hospital, Capital Medical University, Beijing Institute of Heart Lung and Blood Vessel Disease, Beijing Key Laboratory of Precision Medicine of Coronary Atherosclerotic Disease, Clinical Center for Coronary Heart Disease, Capital Medical University, Beijing, 100029 China

**Keywords:** Glycated albumin, Non-ST-segment elevation acute coronary syndrome, Percutaneous coronary intervention, Prognosis

## Abstract

**Background:**

It has been demonstrated that glycated albumin (GA) is significantly associated with diabetes complications and mortality. However, among patients diagnosed with non-ST-elevation acute coronary syndrome (NSTE-ACS) administered percutaneous coronary intervention (PCI), the predictive value of GA for poor prognosis is unclear.

**Methods:**

This study eventually included 2247 NSTE-ACS patients in Beijing Anzhen Hospital, Capital Medical University in January-December 2015 who received PCI. All patients were followed up until death or for 48 months post-discharge. The primary endpoint was major adverse cardio-cerebral events (MACCEs), including all-cause death, non-fatal myocardial infarction, ischemia-induced revascularization and non-fatal ischemic stroke.

**Results:**

In total, 547 (24.3%) MACCEs were recorded during the follow-up period. Upon adjusting for potential confounders, GA remained an important risk predictor of MACCEs (As nominal variate: hazard ratio [HR] 1.527, 95% confidence interval [CI] 1.236–1.886, P < 0.001; As continuous variate: HR 1.053, 95% CI 1.027–1.079, P < 0.001). GA addition significantly enhanced the predictive ability of the traditional risk model (Harrell’s C-index, GA vs. Baseline model, 0.694 vs. 0.684, comparison P = 0.002; continuous net reclassification improvement (continuous-NRI) 0.085, P = 0.053; integrated discrimination improvement (IDI) 0.007, P = 0.020).

**Conclusion:**

GA is highly correlated with poor prognosis in NSTE-ACS patients undergoing PCI, suggesting that it may be a major predictive factor of adverse events among these individuals.

**Supplementary Information:**

The online version contains supplementary material available at 10.1186/s12933-022-01446-3.

## Introduction

Type 2 diabetes mellitus (T2DM) independently and significantly predicts atherosclerotic cardiovascular disease (ASCVD), and increases ASCVD risk by about 2 times [1]. Patients with T2DM also suffer from many risk factors, including dyslipidemia and hypertension, which further increase the risk of ASCVD [2]. Fasting blood glucose (FBG) levels and glycosylated hemoglobin (HbA1c) amounts are widely considered important indicators of blood glucose control. Studies have confirmed that HbA1c can predict coronary artery disease (CAD) severity as well as adverse prognosis [3–5]. Among non-diabetic patients hospitalized with acute coronary syndrome (ACS), FBG ≥ 10 mmol/L could predict one-year mortality [6]. Elevated FBG levels significantly increase 6-month mortality in patients with ACS [7]. However, the constant change of FBG levels over time makes it difficult to accurately predict the risk of disease. Similarly, HbA1c has many limitations in short-term regulation of blood glucose as well as in individuals with large blood glucose fluctuations, chronic kidney disease and/or liver cirrhosis and hemoglobin lesions [8].

In recent years, glycated albumin (GA) has attracted widespread attention for being unaffected by food intake and red blood cell lifespan. GA generally reflects the status of blood sugar control in 2–4 weeks. In cases for whom FBG and HbA1c have the above limitations and cannot accurately reflect the patient's blood glucose levels, GA would be a good surrogate indicator [9]. Glycated serum albumin has 85 glycosylation sites, while HbA1c has only one [10]. According to previous reports, the glycation rate of GA is approximately 4.5 times that of HbA1c [11]. In addition, the GA test is cheaper and faster than HbA1c assessment [12]. More importantly, the half-life of GA is only 12–21 days, and GA testing can provide information about blood sugar control for about 2–3 weeks [13–15]. Therefore, when short-term assessment of blood glucose status is required, e.g., for the adjustment of hypoglycemic therapy during hospitalization, GA is better than HbA1c. At present, GA has been confirmed to be closely related to CAD, ischemic stroke, heart failure, cardiovascular death and other diseases [16]. More interestingly, it was shown serum GA represents a better marker compared with HbA1c for evaluating the presence of CAD, assessing CAD severity and predicting major adverse cardiovascular events [17].

However, the prognostic value of GA in individuals with non-ST-elevation acute coronary syndrome (NSTE-ACS) administered percutaneous coronary intervention (PCI) is largely undefined. In addition, studies comparing the predictive values of FBG, HbA1c and GA in poor cardiovascular prognosis are lacking. Therefore, the current work aimed to assess GA for its predictive value for poor outcomes in NSTE-ACS patients after PCI.

## Materials and methods

### Patients

**Fig. 1:**
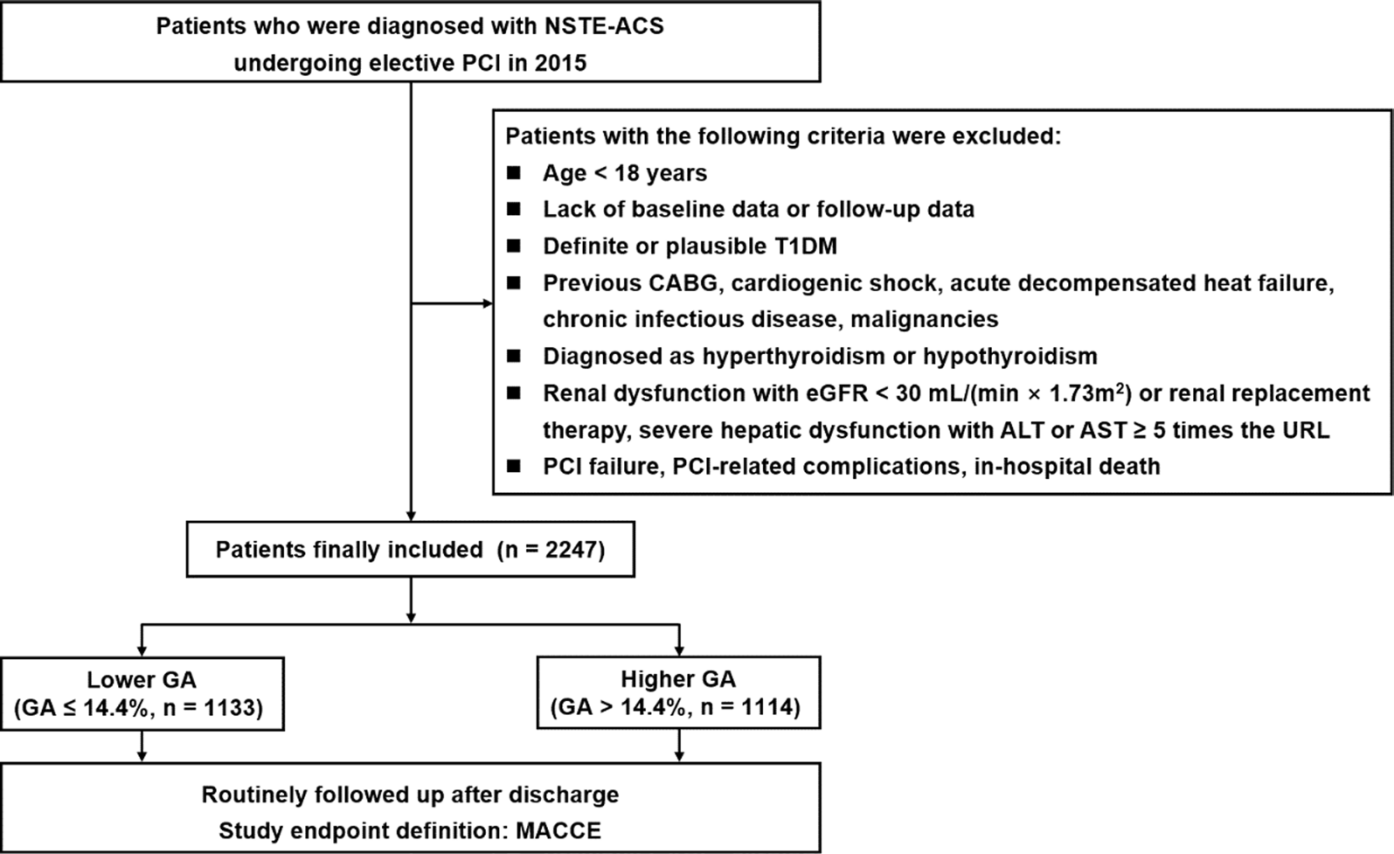
Flow diagram for the enrollment of study population. *NSTE-ACS* non-ST-segment elevation acute coronary syndrome, *PCI* percutaneous coronary intervention, *T1DM* Type 1 Diabetes mellitus, *CABG* coronary artery bypass grafting, *eGFR* estimated glomerular filtration rate, *ALT* alanine transaminase, *AST* aspartate transaminase, *URL* upper reference limit, *GA* glycated albumin, *MACCE* major adverse cardio-cerebral events

This single-center, observational trial continuously included NSTE-ACS cases administered PCI from Jan. to Dec. 2015 in Beijing Anzhen Hospital, Capital Medical University. Diagnostic criteria for NSTE-ACS (including non-ST-segment elevation myocardial infarction [NSTEMI] and unstable angina [UA]) were based on relevant guidelines [18]. Exclusion criteria were: (1) < 18 years of age; (2) lack of baseline or follow-up data; (3) definite or plausible type 1 diabetes mellitus (T1DM); (4) previous coronary artery bypass grafting (CABG), cardiogenic shock, acute decompensated heat failure, chronic infectious disease or malignancy; (5) hyperthyroidism or hypothyroidism; (6) kidney damage (estimated glomerular filtration rate [eGFR] below 30 mL/(min × 1.73 m^2^) or kidney replacement treatment, severe liver dysfunction (alanine or aspartate transaminase amounts ≥ 5 times the upper reference limits); (7) PCI failure, PCI-associated complications or in-hospital death. Finally, totally 2247 individuals were included in this study (Fig. 1).

### Data collection and definitions

Patient baseline data were obtained from the electronic medical information recording system of Beijing Anzhen Hospital. Hypertension was defined as systolic blood pressure (BP) ≥ 140 mmHg and/or diastolic BP ≥ 90 mmHg after repeated measurements on different days [19]. Criteria for T2DM were blood glucose levels ≥ 11.1 mM, FBG ≥ 7.0 mM, and/or 2-h blood glucose after oral glucose tolerance test ≥ 11.1 mM [20, 21]. Dyslipidemia referred to fasting total cholesterol (TC) levels > 200 mg/dL, low-density lipoprotein cholesterol (LDL-C) > 130 mg/dL, triglyceride (TG) levels > 150 mg/dL, high-density lipoprotein cholesterol (HDL-C) < 40 mg/dL and/or long-term administration of lipid-lowering agents. Stroke referred to cerebral infarction or transient ischemic attack. The following conditions were considered peripheral arterial diseases (PADs): non-coronary aortic and arterial-related vascular disease with exercise-associated continuous claudication, decreased or absent pulsation and lumen stenosis of more than 50%.

On the early morning of the operation day, blood samples with fasting time of 8–12 h were taken and sent to the laboratory of the testing center for examination immediately. The GA levels were determined by the enzymatic method using Lucica GA-L kit (Asahi Kasei Pharma, Tokyo) [22]. The value of GA is represented by the percentage of GA concentration in total albumin concentration.

Echocardiograms were verified by 2 ultrasound specialists. Coronary angiography, percutaneous coronary intervention and perioperative management were based on current guidelines [23]. Chronic total occlusion (CTO) was reflected by complete coronary artery occlusion, with Thrombolysis in Myocardial Infarction Flow grade 0 for ≥ 3 months [24]. Complete revascularization was reflected by PCI or bypass of the totality of epicardial vessels with a diameter above 1.5 mm and a luminal reduction above 50% in angiographic views [25].

### Follow-up and study endpoint

After discharge from the hospital, all patients were followed up until death or 48 months after discharge. The primary endpoint was major adverse cardio-cerebral events (MACCEs), including all-cause death, non-fatal myocardial infarction (MI), non-fatal ischemic stroke and ischemia-induced revascularization. MI was reflected by increased cardiac troponin or creatine kinase levels surpassing the upper limits of the reference ranges, with ischemia signs and/or electrocardiogram findings suggesting myocardial ischemia. Stroke definition involved signs of neurological damage, caused by ischemic lesions confirmed by computed tomography or magnetic resonance imaging. Ischemia-induced revascularization was reflected by revascularization in target and/or non-target vessels due to recurring or persistent ischemic symptoms, including PCI and CABG.

### Statistical analysis

Cases were assigned to 2 groups based on median GA (lower GA [GA < 14.4], higher GA [GA ≥ 14.4]). Normally distributed continuous variates are mean ± standard deviation, and were compared by two-sample independent t test. Continuous variates with skewed distribution were represented by median and 25th and 75th percentiles, and compared by the Mann–Whitney U test. Nominal variates were described by numbers and percentages, and compared by the Chi-square, continuity-corrected chi-square or Fisher’s exact test.

The Kaplan–Meier method was utilized for describing event rates at follow-up and plotting time-to-event curves in both groups, which were compared by the log rank test. The univariable Cox proportional hazards model was used for preliminary assessment of factors associated with MACCEs. Variates with significant associations with MACCEs and those that may be meaningful based on clinical experience were included in five multivariate models. Variates with potential collinearity were not included in the multivariate analysis. GA was tested as nominal and continuous variables, respectively. Hazard ratios (HRs) and 95% confidence intervals (CIs) were used to describe the associations. In multivariable Cox proportional hazard analysis, five models were established for evaluating GA’s predictive value in MACCEs: Model 1, adjustment for age, gender and body mass index (BMI); Model 2, adjustment for Model 1 variables and smoking history, hypertension, T2DM, anemia and previously diagnosed MI, PCI and stroke; Model 3, adjustment for Model 2 variables and TG, TC, creatinine, high-sensitivity C-reactive protein (hs-CRP), HDL-C, left ventricular ejection fraction (LVEF); Model 4, adjustment for Model 3 variables and oral hypoglycemic agent (OHA) and insulin prescriptions at discharge; Model 5, adjustment for Model 4 variables and left main artery (LM) lesion, multi-vessel lesion, SYNTAX score, complete revascularization and drug-eluting stent (DES) amount. According to Model 5, a restrictive cubic spline curve was established to illustrate the dose–response association of GA with MACCEs. Except for variables used for stratification, stratified analysis adjusted for Model 5 variables. Interactions were examined by the likelihood ratio test.

Harrell’s C-index, net reclassification improvement (NRI) and integrated discrimination improvement (IDI) were used for investigating the additive effects of GA on the predictive abilities of traditional cardiovascular disease risk factors in MACCEs.

SPSS v26.0 and R v3.6.3 were used for data analysis. Two-tailed *P* < 0.05 was deemed statistically significant.

## Results

### Baseline patient features

Totally 2247 patients were included, with an average age of 60.1 ± 9.0, and the proportion of males was 71.9% (n = 1616). Patients were assigned to 2 groups based on median GA. Demographic data, clinical features, laboratory results, and medical and procedural details are shown in Tables 1 and 2. In the high GA group, participants were older and had a lower proportion of men compared with the low GA group. Participants with high GA levels had higher heart rate, systolic blood pressure and incidence rates of hypertension and T2DM, and lower rates of smoking and drinking history. Higher rates of previous PCI and previous stroke were observed in individuals with high GA. For laboratory examinations, participants with high GA had lower levels of TC, LDL-C, creatinine and uric acid, while FBG and HbA1c amounts were elevated. Regarding medication at admission, patients with higher GA received a higher proportion of OHA and insulin treatments, and a lower proportion of statins. In terms of discharge medications, participants with high GA were prescribed angiotensin-converting enzyme inhibitors (ACEIs)/angiotensin receptor blockers (ARBs), OHA and insulin at a higher rate. Regarding coronary angiography and PCI, in the GA high group, the proportions of multivessel lesion and in-stent restenosis were higher. Participants with high GA had more target vessels of left circumflex artery (LCX) and right coronary artery (RCA) treated, more DES implanted, and a lower proportion of complete revascularization.

**Table 1:** Baseline demographic, clinical and laboratory characteristics of the study population

	Total population (n = 2247)	Lower GA (≤ 14.4%, n = 1133)	Higher GA (> 14.4%, n = 1114)	*P* value
Age, years	60.1 ± 9.0	58.2 ± 9.2	62.0 ± 8.3	< 0.001
Gender, male, n (%)	1616 (71.9)	864 (76.3)	752 (67.5)	< 0.001
BMI, kg/m^2^	26.1 ± 3.2	26.2 ± 3.2	26.0 ± 3.2	0.323
Heart rate, bpm	69.7 ± 10.2	68.9 ± 9.6	70.6 ± 10.6	< 0.001
SBP, mmHg	130.2 ± 16.5	128.9 ± 15.9	131.6 ± 16.9	< 0.001
DBP, mmHg	77.0 ± 9.8	77.3 ± 9.3	76.7 ± 10.2	0.162
Smoking history, n (%)	1280 (57.0)	714 (63.0)	566 (50.8)	< 0.001
Drinking history, n (%)	526 (23.4)	300 (26.5)	226 (20.3)	0.001
Family history of CAD, n (%)	233 (10.4)	120 (10.6)	113 (10.1)	0.728
Medical history, n (%)				
T2DM	774 (34.4)	101 (4.5)	673 (30.0)	< 0.001
Hypertension	1397 (62.2)	671 (59.2)	726 (65.2)	0.004
Hyperlipidemia	1932 (86.0)	979 (86.4)	953 (85.5)	0.557
Anemia	33 (1.5)	8 (0.7)	25 (2.2)	0.002
Previous MI	473 (21.1)	220 (19.4)	253 (22.7)	0.056
Previous PCI	376 (16.7)	161 (14.2)	215 (19.3)	0.001
Previous stroke	259 (11.5)	113 (10.0)	146 (13.1)	0.020
Previous PAD	79 (3.5)	36 (3.2)	43 (3.9)	0.380
Clinical diagnosis, n (%)				
UA	1873 (83.4)	951 (83.9)	922 (82.8)	0.456
NSTEMI	374 (16.6)	182 (16.1)	192 (17.2)	
Laboratory examinations				
TG, mmol/L	1.5 (1.1, 2.1)	1.5 (1.1, 2.1)	1.4 (1.0, 2.1)	0.440
TC, mmol/L	4.1 ± 1.0	4.2 ± 1.0	4.1 ± 1.0	0.029
LDL-C, mmol/L	2.5 ± 0.9	2.5 ± 0.9	2.5 ± 0.8	0.022
HDL-C, mmol/L	1.0 ± 0.2	1.0 ± 0.2	1.0 ± 0.2	0.261
hs-CRP, mg/L	1.3 (0.6, 3.3)	1.2 (0.5, 2.9)	1.3 (0.6, 3.8)	0.006
Creatinine, μmol/L	75.8 ± 16.5	76.9 ± 16.7	74.7 ± 16.3	0.001
eGFR, mL/(min × 1.73 m^2^)	93.6 ± 20.0	93.7 ± 19.5	93.5 ± 20.5	0.790
Uric acid, μmol/L	344.1 ± 80.4	358.6 ± 79.5	329.5 ± 78.6	0.001
FBG, mmol/L	6.1 ± 1.9	5.3 ± 0.9	6.9 ± 2.3	< 0.001
HbA1c, %	6.3 ± 1.2	5.7 ± 0.5	6.9 ± 1.4	0.001
LVEF, %	64.0 ± 6.7	63.9 ± 7.0	64.0 ± 6.5	0.625

*GA* glycated albumin, *BMI* body mass index, *SBP* systolic blood pressure, *DBP* diastolic blood pressure, *CAD* coronary artery disease, *T2DM* type 2 diabetes mellitus, *MI* myocardial infarction, *PCI* percutaneous coronary intervention, *PAD* peripheral artery disease, *UA* unstable angina, *NSTEMI* non-ST-segment elevation myocardial infarction, *TG* triglyceride, *TC* total cholesterol, *LDL-C* low-density lipoprotein cholesterol, *HDL-C* high-density lipoprotein cholesterol, *hs-CRP* high-sensitivity C-reactive protein, *eGFR* estimated glomerular filtration rate, *FBG* fasting blood glucose, *HbA1c* glycosylated hemoglobin A1c, *LVEF* left ventricular ejection fraction

**Table 2:** Therapeutic, angiographic, and procedural characteristics of the study population

	Total population (n = 2247)	Lower GA (≤ 14.4%, n = 1133)	Higher GA (> 14.4%, n = 1114)	*P* value
Medication at admission, n (%)				
ACEI/ARB	500 (22.3)	246 (21.7)	254 (22.8)	0.535
DAPT	677 (30.1)	348 (30.7)	329 (29.5)	0.542
Aspirin	1192 (53.0)	598 (52.8)	594 (53.3)	0.797
P2Y12 inhibitors	718 (32.0)	371 (32.7)	347 (31.1)	0.417
β-Blocker	496 (22.1)	251 (22.2)	245 (22.0)	0.927
Statins	691 (30.8)	370 (32.7)	321 (28.8)	0.048
OHA	400 (17.8)	56 (4.9)	344 (30.9)	< 0.001
Insulin	218 (9.7)	13 (1.1)	205 (18.4)	< 0.001
Medication at discharge, n (%)				
ACEI/ARB	1558 (69.3)	758 (66.9)	800 (71.8)	0.012
DAPT	2245 (99.9)	1133(100.0)	1112 (99.8)	0.154
Aspirin	2246 (100.0)	1133 (100.0)	1113(99.9)	0.313
P2Y12 inhibitors	2247 (100.0)	1133 (100.0)	1114 (100.0)	–
β-Blocker	2045 (91.0)	1024 (90.4)	1021 (91.7)	0.292
Statins	2195 (97.7)	1101 (97.2)	1094 (98.2)	0.105
OHA	396 (17.6)	56 (4.9)	340 (30.5)	< 0.001
Insulin	211 (9.4)	12 (1.1)	199 (17.9)	< 0.001
Angiographic data, n (%)				
LM lesion	102 (4.5)	45 (4.0)	57 (5.1)	0.192
Multi-vessel lesion	1498 (66.7)	655 (57.8)	843 (75.7)	< 0.001
In-stent restenosis	124 (5.5)	47 (4.1)	77 (6.9)	0.004
Chronic total occlusion lesion	295 (13.1)	136 (12.0)	159 (14.3)	0.111
SYNTAX score	11.0 ± 5.4	10.0 ± 5.1	12.0 ± 5.5	< 0.001
Procedural information				
Target vessel territory, n (%)				
LM	60 (2.7)	31 (2.7)	29 (2.6)	0.845
LAD	1464 (65.2)	738 (65.1)	726 (65.2)	0.987
LCX	784 (34.9)	364 (32.1)	420 (37.7)	0.006
RCA	952 (42.4)	434 (38.3)	518 (46.5)	< 0.001
Complete revascularization, n (%)	1323 (58.9)	746 (65.8)	577 (51.8)	< 0.001
Number of DES	2.0 ± 1.3	1.9 ± 1.3	2.0 ± 1.3	0.022

*GA* glycated albumin, *ACEI* angiotensin-converting enzyme inhibitor, *ARB* angiotensin receptor blocker, *DAPT* dual antiplatelet therapy, *OHA* oral hypoglycemic agents, *LM* left main artery, *SYNTAX* synergy between PCI with taxus and cardiac surgery *LAD* left anterior descending artery, *LCX* left circumflex artery, *RCA* right coronary artery, *DES* drug-eluting stent

### Predictive value of GA for MACCE

After 48 months of follow-up, 547 (24.3%) cases of MACCEs were recorded, including 36 (1.6%) all-cause death, 112 (5.0%) non-fatal myocardial infarction (MI), 45 (2.0%) non-fatal stroke and 354 (15.8%) ischemia-driven revascularization cases. The incidence rates of MACCEs (*P* < 0.001), all-cause death (*P* = 0.006), non-fatal MI (*P* = 0.001) and ischemia-driven revascularization (*P* < 0.001) were significantly higher in the high GA group compared with the low GA group. However, the incidence rates of non-fatal stroke were comparable in both groups (Table 3).

**Table 3:** Incidence of primary endpoint and each component according to the median of GA

	Total population (n = 2247)	Lower GA (≤ 14.4%, n = 1133)	Higher GA (> 14.4%, n = 1114)	*P* value
MACCE, n (%)	547 (24.3)	205 (18.1)	342 (30.7)	< 0.001
All-cause death, n (%)	36 (1.6)	10 (0.9)	26 (2.3)	0.006
Non-fatal MI, n (%)	112 (5.0)	40 (3.5)	72 (6.5)	0.001
Non-fatal ischemic stroke, n (%)	45 (2.0)	22 (1.9)	23 (2.1)	0.835
Ischemia-driven revascularization, n (%)	354 (15.8)	133 (11.7)	221 (19.8)	< 0.001

*GA* glycated albumin, *MACCE* major adverse cardio-cerebral events, *MI* myocardial infarction

**Fig. 2:**
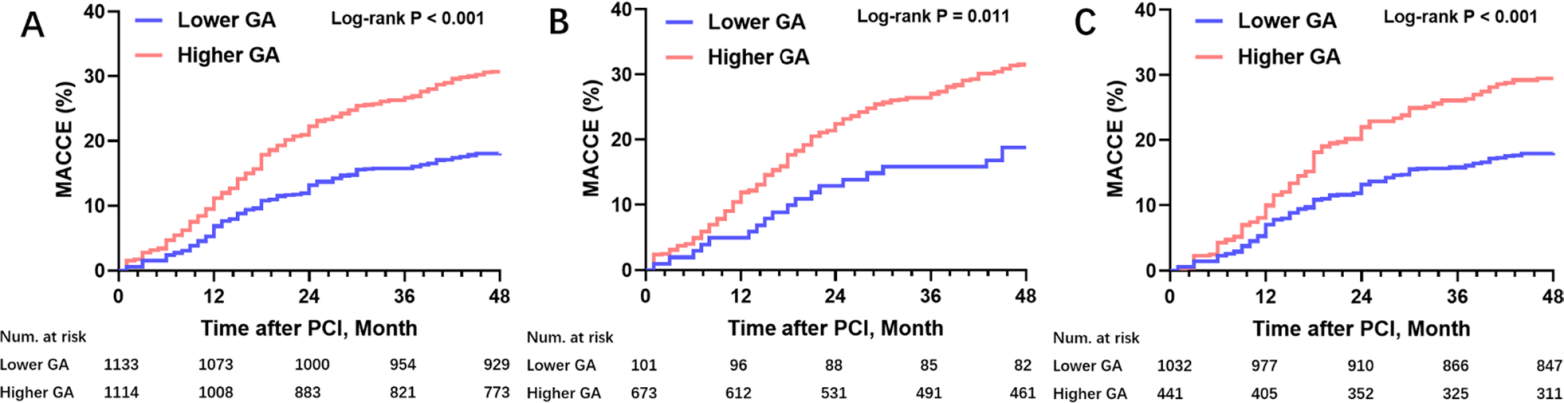
Kaplan–Meier survival curves according to the median of GA. **A** Kaplan–Meier survival curves for the primary endpoint in the entire population; **B** Kaplan–Meier survival curves for the primary endpoint in the patients with T2DM; **C** Kaplan–Meier survival curves for the primary endpoint in the patients without T2DM. *GA* glycated albumin, *MACCE* major adverse cardio-cerebral events

Kaplan–Meier analysis was performed for evaluating the time-dependent cumulative incidence of MACCEs collectively and individually in both groups in the general, diabetic and non-diabetic populations. In the general population, the cumulative incidence of MACCEs was increased significantly in the high GA group in comparison with the low GA group (Fig. 2A, log-rank P < 0.001). Similar results were obtained in diabetic (Fig. 2B, log-rank P = 0.011) and non-diabetic (Fig. 2C, log-rank P < 0.001) populations.

Furthermore, five multivariate models were established for assessing the predictive performances of GA for MACCEs. Univariate Cox proportional hazards analysis was used to initially define the potential determinants of the primary endpoint (Additional file 1: Table S1). According to univariate analysis (P < 0.05) and clinical importance, variables were included in the multivariate models (shown in Methods). After adjusting for variates in the five models, whether GA was considered a nominal or continuous variable, it showed significant independent prognostic value in all models (Table 4).

**Table 4:** Predictive value of GA for the risk of MACCE

	As nominal variate^a^		As continuous variate^b^	
	HR (95% CI)	*P* value	HR (95% CI)	*P* value
Unadjusted	1.826 (1.536–2.171)	< 0.001	1.072 (1.054–1.091)	< 0.001
Model 1	1.639 (1.374–1.956)	< 0.001	1.065 (1.046–1.083)	< 0.001
Model 2	1.510 (1.226–1.858)	< 0.001	1.063 (1.039–1.088)	< 0.001
Model 3	1.610 (1.304–1.987)	< 0.001	1.063 (1.038–1.089)	< 0.001
Model 4	1.602 (1.297–1.979)	< 0.001	1.062 (1.036–1.088)	< 0.001
Model 5	1.527 (1.236–1.886)	< 0.001	1.053 (1.027–1.079)	< 0.001

Model 1: adjusted for age, gender, BMI

Model 2: adjusted for variates in Model 1 and smoking history, hypertension, T2DM, anemia, previous MI, previous PCI, previous stroke

Model 3: adjusted for variates in Model 2 and TG, TC, creatinine, hs-CRP, HDL-C, LVEF

Model 4: adjusted for variates in Model 3 and OHA at discharge, insulin at discharge

Model 5: adjusted for variates in Model 4 and left main artery lesion, multi-vessel lesion, SYNTAX score, complete revascularization, number of DES

^a^The HR was evaluated regarding the lower median of GA as reference

^b^The HR was evaluated by per 1-unit increase of GA

*HR* hazard ratio, *CI* confidence interval

**Fig. 3:**
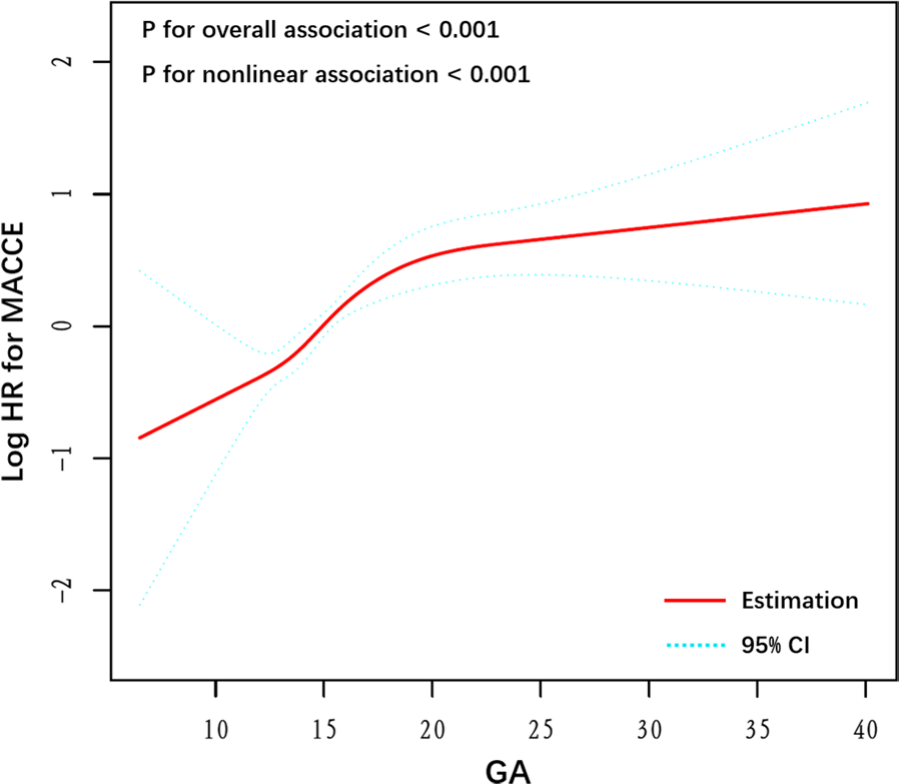
Restricted cubic smoothing for the risk of the primary endpoint according to the GA. The analysis was adjusted for Model 5. HR was evaluated by per 1-unit increase of GA. *GA* glycated albumin, *MACCE* major adverse cardio-cerebral events

After adjusting for variates in Model 5, the dose–response relationship between GA level and MACCEs was illustrated by drawing restricted cubic spline curve (Fig. 3). It was found that MACCE risk increased with GA level (*P* for overall association < 0.001), suggesting that GA had a linear relationship with MACCE risk. This was further confirmed in the non-linear correlation test (*P* for nonlinear association < 0.001).

**Fig. 4:**
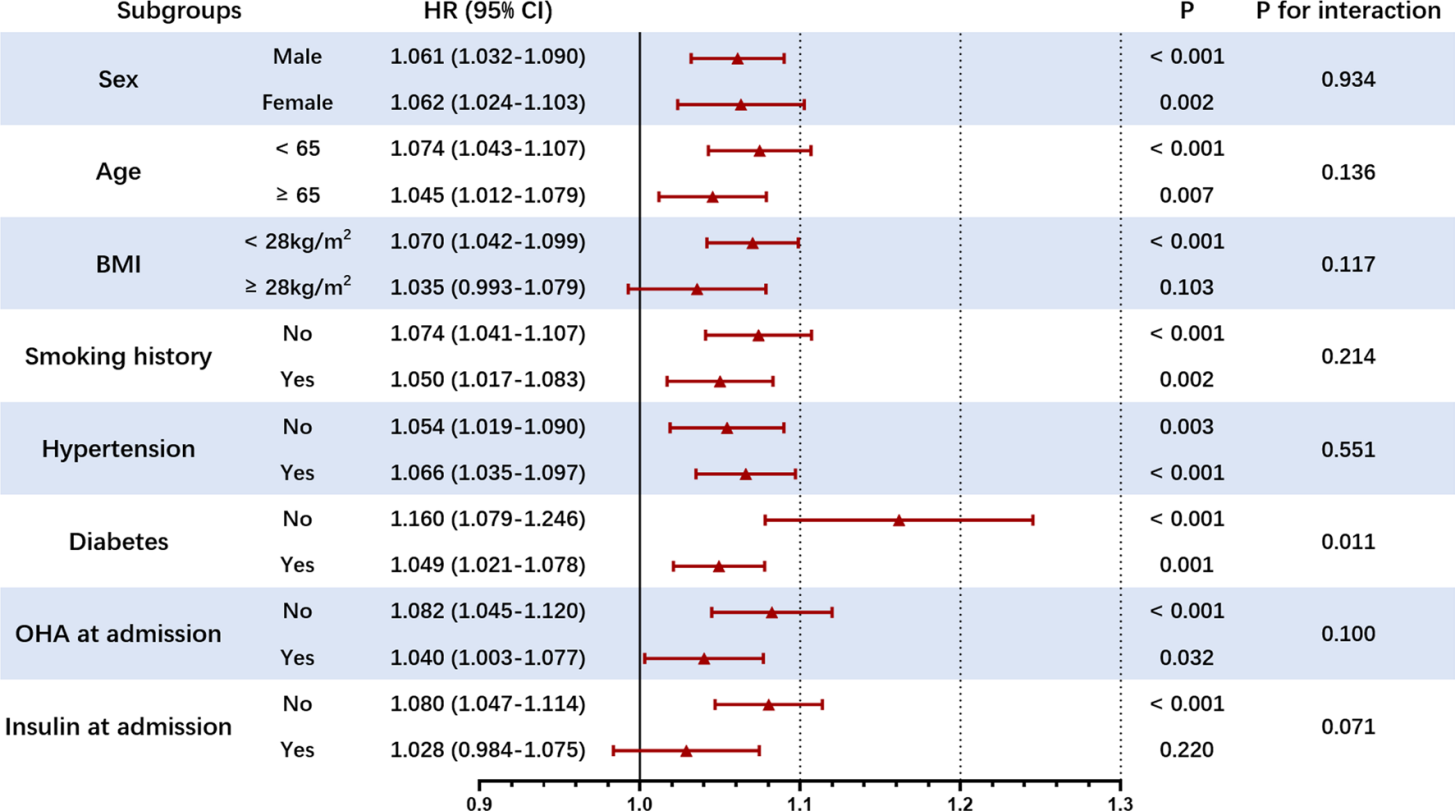
Subgroup analysis evaluating the robustness of GA in predicting the risk of the primary endpoint. The analysis was adjusted for Model 5 except for variates applied for grouping. HR was evaluated by per 1-unit increase of GA. *BMI* body mass index, *OHA* oral hypoglycemic agents

Subgroup analysis further confirmed the predictive value of GA for MACCEs. In the subgroups of gender (male or female), age (< 65 or ≥ 65 years), BMI (< 28 or ≥ 28 kg/m^2^), smoking history (no or yes), hypertension (no or yes), OHA at admission (no or yes), and insulin at admission (no or yes), there were no differences in the predictive power of GA in MACCEs (all *P* for interaction > 0.05). It is worth noting that the predictive value of GA seemed to be higher in non-diabetic patients [HR (95%CI) T2DM no 1.167 (1.017–1.087) vs. T2DM yes 1.047 (1.019–1.075), *P* for interaction = 0.006] (Fig. 4).

### GA increases the predictive values of other factors for MACCEs

In the baseline model comprising the currently known cardiovascular risk factors (age, gender, BMI, smoking history, family history of CAD, hypertension, T2DM, anemia, NSTEMI, creatinine, TC, LVEF, LM lesion, multi-vessel lesion and SYNTAX score), addition of GA markedly enhanced the ability of the model to predict risk (Harrell’s C-index: GA vs. Baseline model, 0.694 vs. 0.684, *P* = 0.002). The reclassification and discrimination abilities were significantly improved in comparison with the baseline risk model after addition of GA (Continuous-NRI = 0.085, *P* = 0.053; IDI = 0.007, *P* = 0.020). In Harrell’s C-index, NRI and IDI analysis, addition of FBG (Harrell’s C-index: FBG, 0.692 vs. baseline risk model, 0.684, *P* = 0.001; Continuous NRI: 0.087, P = 0.053; IDI: 0.005, P = 0.040) and HbA1c (Harrell’s C-index: HbA1c, 0.693 vs. baseline risk model, 0.684, *P* = 0.005; Continuous NRI: 0.048, P = 0.106; IDI: 0.006, P = 0.033) also significantly improved the risk prediction ability of the baseline model (Table 5). Although it seems that GA is not better than FBG and HbA1c in improving the predictive ability of the baseline model, it is not inferior to the latter two either. The parameters for each variate in these models are displayed in Additional file 1: Table S2–S5.

**Table 5:** Incremental effects of GA, FBG, and HbA1c on risk stratification for the MACCE beyond existing risk factors

	Chi-square value	Harrell’s C-index			Continuous-NRI			IDI		
		Estimation	95% CI	P for comparison	Estimation	95% CI	*P* value	Estimation	95% CI	*P* value
Baseline model	235.533	0.684	0.663–0.706	–	–	–	–	–	–	–
+ GA	260.704	0.694	0.673–0.715	0.002	0.085	− 0.004–0.138	0.053	0.007	0.001–0.017	0.020
+ FBG	257.774	0.692	0.671–0.713	0.001	0.087	− 0.002–0.144	0.053	0.005	0.000–0.013	0.040
+ HbA1c	258.198	0.693	0.672–0.714	0.005	0.048	− 0.012–0.103	0.106	0.006	0.001–0.014	0.033

The baseline model included age, gender, BMI, smoking history, family history of CAD, hypertension, T2DM, anemia, NSTEMI, creatinine, TC, LVEF, LM lesion, multi-vessel lesion and SYNTAX score

*NRI* net reclassification improvement, *IDI* integrated discrimination improvement, *CI* confidence interval

*GA* glycated albumin, *FBG* fasting blood glucose, *HbA1c* glycosylated hemoglobin A1c

## Discussion

The present work firstly assessed the predictive value of GA for poor prognosis in NSTE-ACS patients after PCI. We found that the incidence of MACCEs was markedly elevated in individuals with high GA levels in comparison with the low GA group. Upon adjustment for confounding factors, GA increase was still an important and independent predictor of poor prognosis in the study population. Adding GA to the model comprising traditional risk factors significantly improved its ability to predict the risk of poor prognosis.

40 years ago, researchers firstly found elevated GA levels in the serum of diabetic patients [26]. Then, with studies assessing GA test methods and comparative assessment of GA and HbA1c, GA has gradually been used as a marker of diabetes in clinical practice [27–29]. In patients with T1DM and T2DM administered hypoglycemic therapy, the change in GA at 4 weeks is the same as that of HbA1c at 12 weeks [30]. GA can not only reflect short-term average blood glucose, but also indicate blood glucose fluctuations. Compared with HbA1c, GA has more obvious advantages with rapid changes in blood sugar or rapid deterioration of blood glucose [31], such as in fulminant type I diabetes. GA can also monitor postprandial blood glucose’s swimming fluctuations and hypoglycemia as well as other pathologic factors [32, 33].

Many studies have also explored the value of GA in ASCVD. Based on Atherosclerosis Risk in Communities (ARIC) Study in 1990–1992, Selvin et al. followed up 11,104 patients for 20 years, and found that GA was associated with vascular outcomes and mortality in the community, and these associations were similar to those observed for HbA1c [16]. HbA1c, GA and FBG levels are positively correlated with carotid artery intima-media thickness, which is widely considered an early sign of atherosclerosis [34]. In patients receiving PCI, Yang et al. tested serum GA in 576 type 2 diabetes and stable CAD cases who were implanted with a sirolimus-eluting stent. After two years of follow-up and adjustment for possible confounding factors, serum GA level (HR = 1.22, 95% CI 1.16–1.28; HR = 1.15, 95% CI 1.11–1.19, respectively; both P < 0.001) still independently predicted the primary (cardiac death, non-fatal myocardial infarction and non-fatal stroke) and secondary (occurrence of clinically driven repeat revascularization) outcomes [35]. In addition, studies have also confirmed that GA level increase is highly correlated with the severity of coronary artery damage in T2DM and CAD cases [36, 37], as well as impaired collateral growth in patients with CTO [38]. Combined with the above studies, our results further clarify the predictive value of elevated GA for poor prognosis in NSTE-ACS patients undergoing PCI, and the results were consistent with previous conclusions. Multivariate and subgroup analyses in this study showed that GA is significant and robust as a predictor of adverse cardiovascular and cerebrovascular events. Interestingly, however, GA showed higher predictive value in the non-diabetic subgroup compared with the diabetic subgroup. A study involving 2965 Japanese community residents aged ≥ 40 years with a median follow-up time of 10.2 years confirmed that the increase in GA levels were significantly associated with the development of cardiovascular disease, even in the general population without diabetes [39]. It suggested that before the onset of diabetes, the increase in serum GA levels is closely related to the occurrence and development of cardiovascular disease. GA may have the potential as a routine examination for patients with cardiovascular disease. However, it still needs to be further confirmed with large-scale prospective studies. HbA1c has been demonstrated to be an independent predictor of CAD odds and severity in non-diabetic individuals [40]. Therefore, comparing the predictive value of GA and HbA1c for the prognosis of patients with non-diabetic cardiovascular disease is a meaningful research direction. On the other hand, although addition of GA seems to improve the ability of traditional risk models to predict poor prognosis, GA did not show more advantages than FBG and HbA1c in this study.

Regarding the mechanism-level explanation of GA’s predictive value for poor prognosis in atherosclerotic cardiovascular disease, inflammation has attracted widespread attention. In cultured rat vascular smooth muscle cells (VSMCs), GA can induce the expression of the pro-inflammatory cytokine IL-6 at the mRNA level [41]. The presence of GA is harmful to endothelial cells, which become more pro-coagulant, promoting inflammation [42]. GA’s ability to predict poor prognosis in atherosclerotic cardiovascular disease may also have other mechanisms. Du and collaborators confirmed elevated serum GA amounts are associated with negative coronary artery remodeling in type 2 diabetes cases [43]. In addition, Rubenstein et al. found that the presence of GA enhances platelet aggregation, with the degree of glycation enhancing platelet activation [44]. Yamada et al. found that GA is highly correlated with peripheral vascular calcification in type 2 diabetic hemodialysis [45]. In summary, the role of GA in cardiovascular atherosclerosis may involve multiple pathophysiological processes.

There were limitations in this study. First, this was a single-center, retrospective, observational trial, which might reduce the effectiveness and power of these research findings. Therefore, more in-depth prospective, multi-center studies are required to further verify the current findings. Secondly, some patients received anti-diabetic treatment before admission, which may have affected the actual level of GA. Thirdly, factors such as age, obesity, inflammation, etc. may impact GA levels in this work. Fourthly, this study only included Chinese patients, and the generalizability of the findings to other ethnicities requires further investigation. Fifth, in our research population, patients with UA account for the vast majority. Therefore, the predictive value of GA on the prognosis of patients with NSTEMI may not be well shown.

## Conclusions

In NSTE-ACS patients administered PCI, GA level is significantly correlated with high risk of adverse cardio-cerebral events. Addition of GA significantly improves the ability of traditional risk models to predict poor prognosis. This conclusion needs further prospective, large-scale studies for confirmation.

## Supplementary Information

**Additional file 1.** Additional table.

## Data Availability

The datasets used during the current study are available from the corresponding author on reasonable request.

## Abbreviations

T2DM: Type 2 diabetes mellitus
ASCVD: Atherosclerotic cardiovascular disease
FBG: Fasting blood glucose
HbA1c: Glycosylated hemoglobin
CAD: Coronary artery disease
ACS: Acute coronary syndrome
GA: Glycated albumin
NSTE-ACS: Non-ST-segment elevation acute coronary syndrome
PCI: Percutaneous coronary intervention
NSTEM: Non-ST-segment elevation myocardial infarction
UA: Unstable angina
T1DM: Type 1 diabetes mellitus
CABG: Coronary artery bypass grafting
eGFR: Estimated glomerular filtration rate
BP: Blood pressure
TC: Total cholesterol
LDL-C: Low-density lipoprotein cholesterol
TG: Triglyceride
HDL-C: High-density lipoprotein cholesterol
PAD: Peripheral arterial disease
CTO: Chronic total occlusion
MACCE: Major adverse cardio-cerebral event
MI: Myocardial infarction
HR: Hazard ratio
CI: Confidence interval
BMI: Body mass index
hs-CRP: High-sensitivity C-reactive protein
LVEF: Left ventricular ejection fraction
OHA: Oral hypoglycemic agents
LM: Left main artery
DES: Drug-eluting stent
NRI: Net reclassification improvement
IDI: Integrated discrimination improvement
ACEI: Angiotensin-converting enzyme inhibitor
ARB: Angiotensin receptor blocker
LCX: Left circumflex artery
RCA: Right coronary artery
MI: Myocardial infarction
ARIC: Atherosclerosis risk in communities
VSMC: Vascular smooth muscle cell
