# Supplementary material for: Prognostic implication of serum glycated albumin for patients with non-ST-segment elevation acute coronary syndrome undergoing percutaneous coronary intervention
Source: Cardiovasc Diabetol. 2022 Jan 19;21:11. doi: 10.1186/s12933-022-01446-3 (PMC8772172; doi:10.1186/s12933-022-01446-3)
Supplement: Supplementary file 1 — Additional file 1. Additional table. [file 12933_2022_1446_MOESM1_ESM.docx]

## Table S1. Unadjusted Cox regression analysis investigating predictors of primary endpoint

|  | MACCE | | |
| --- | --- | --- | --- |
|  | HR | 95% CI | *P* value |
| Age, per 1 years | 1.036 | 1.025-1.046 | <0.001 |
| Gender, male as reference | 1.227 | 1.025-1.470 | 0.026 |
| BMI, per 1 kg/m^2^ | 1.030 | 1.004-1.057 | 0.023 |
| Heart rate, per 1 bpm | 1.007 | 0.999-1.015 | 0.107 |
| SBP, per 1 mmHg | 1.007 | 1.002-1.012 | 0.008 |
| DBP, per 1 mmHg | 0.999 | 0.991-1.008 | 0.858 |
| Smoking history | 0.916 | 0.774-1.084 | 0.308 |
| Drinking history | 0.843 | 0.686-1.036 | 0.104 |
| Family history of CAD | 1.029 | 0.784-1.351 | 0.836 |
| T2DM | 1.460 | 1.232-1.730 | <0.001 |
| Hypertension | 1.273 | 1.065-1.522 | 0.008 |
| Anemia | 1.255 | 0.671-2.346 | 0.477 |
| Previous MI | 2.269 | 1.902-2.707 | <0.001 |
| Previous PCI | 1.734 | 1.426-2.108 | <0.001 |
| Previous PAD | 1.155 | 0.747-1.787 | 0.517 |
| TG, per 1 mmol/L | 1.571 | 1.455-1.695 | <0.001 |
| TC, per 1 mmol/L | 1.118 | 1.035-1.207 | 0.004 |
| LDL-C, per 1 mmol/L | 1.040 | 0.947-1.141 | 0.415 |
| HDL-C, per 1 mmol/L | 0.334 | 0.225-0.494 | <0.001 |
| hs-CRP, per 1 mg/L | 1.012 | 0.999-1.024 | 0.062 |
| Creatinine, μmol/L | 1.005 | 1.000-1.010 | 0.044 |
| FBG, per 1 mmol/L | 1.116 | 1.084-1.150 | <0.001 |
| HbA1c, per 1% | 1.262 | 1.192-1.336 | <0.001 |
| LVEF, per 1% | 0.962 | 0.951-0.973 | <0.001 |
| DAPT at discharge | 0.443 | 0.062-3.151 | 0.416 |
| Statins at discharge | 1.450 | 0.750-2.802 | 0.269 |
| OHA at discharge | 1.310 | 1.068-1.606 | 0.009 |
| Insulin at discharge | 1.641 | 1.284-2.096 | <0.001 |
| LM lesion | 2.450 | 1.822-3.295 | <0.001 |
| Multi-vessel lesion | 2.936 | 2.337-3.689 | <0.001 |
| Complete revascularization | 0.589 | 0.498-0.696 | <0.001 |
| Number of DES, per 1 DES | 1.257 | 1.187-1.331 | <0.001 |
| SYNTAX score | 1.070 | 1.055-1.086 | <0.001 |

*BMI* body mass index, *SBP* systolic blood pressure, *DBP* diastolic blood pressure, *CAD* coronary artery disease, *T2DM* type 2 diabetes mellitus, *MI* myocardial infarction, *PCI* percutaneous coronary intervention, *PAD* peripheral artery disease, *UA* unstable angina, *TG* triglyceride, *TC* total cholesterol, *LDL-C* low-density lipoprotein cholesterol, *HDL-C* high-density lipoprotein cholesterol, *hs-CRP* high-sensitivity C-reactive protein, *FBG* fasting blood glucose, *HbA1c* glycosylated hemoglobin A1c, *LVEF* left ventricular ejection fraction, *DAPT* dual antiplatelet therapy, *OHA* oral hypoglycemic agents, *LM* left main artery, *DES* drug-eluting stent, *SYNTAX* synergy between PCI with taxus and cardiac surgery

## Table S2. Parameters for the baseline model (sort by Chi square value in descending order)

|  | Chi-square value | β | HR | 95% CI | *P* value |
| --- | --- | --- | --- | --- | --- |
| LVEF | 37.961 | -0.037 | 0.963 | 0.952-0.975 | <0.001 |
| Age | 36.935 | 0.033 | 1.033 | 1.022-1.044 | <0.001 |
| Multi-vessel lesion | 23.559 | 0.660 | 1.934 | 1.482-2.524 | <0.001 |
| LM lesion | 14.117 | 0.614 | 1.848 | 1.342-2.546 | <0.001 |
| TC | 11.362 | 0.134 | 1.144 | 1.058-1.237 | 0.001 |
| SYNTAX score | 7.552 | 0.027 | 1.027 | 1.008-1.047 | 0.006 |
| Gender | 5.119 | 0.269 | 1.309 | 1.037-1.653 | 0.024 |
| Creatinine | 3.820 | 0.005 | 1.005 | 1.000-1.010 | 0.051 |
| T2DM | 3.541 | 0.171 | 1.187 | 0.993-1.419 | 0.060 |
| BMI | 3.184 | 0.024 | 1.024 | 0.998-1.052 | 0.074 |
| Hypertension | 2.054 | 0.137 | 1.147 | 0.951-1.383 | 0.152 |
| Smoking history | 1.001 | 0.102 | 1.107 | 0.907-1.352 | 0.317 |
| NSTEMI | 0.902 | 0.104 | 1.110 | 0.895-1.377 | 0.342 |
| Anemia | 0.656 | 0.261 | 1.298 | 0.690-2.443 | 0.418 |
| Family history of CAD | 0.019 | 0.020 | 1.020 | 0.775-1.342 | 0.889 |

*LVEF* left ventricular ejection fraction, *LM* left main artery, *TC* total cholesterol, *SYNTAX* synergy between PCI with taxus and cardiac surgery, *T2DM* type 2 diabetes mellitus, *BMI* body mass index, *NSTEMI* non-ST-segment elevation myocardial infarction, *CAD* coronary artery disease

## Table S3. Parameters for the baseline + GA model (sort by Chi square value in descending order)

|  | Chi-square value | β | HR | 95% CI | *P* value |
| --- | --- | --- | --- | --- | --- |
| LVEF | 36.145 | -0.037 | 0.964 | 0.953-0.976 | <0.001 |
| Age | 32.936 | 0.031 | 1.031 | 1.021-1.042 | <0.001 |
| Multi-vessel lesion | 23.029 | 0.654 | 1.922 | 1.472-2.511 | <0.001 |
| GA | 20.504 | 0.055 | 1.056 | 1.031-1.081 | <0.001 |
| LM lesion | 13.436 | 0.599 | 1.821 | 1.322-2.508 | <0.001 |
| TC | 11.080 | 0.134 | 1.143 | 1.056-1.236 | 0.001 |
| SYNTAX score | 5.674 | 0.023 | 1.024 | 1.004-1.043 | 0.017 |
| BMI | 4.866 | 0.030 | 1.030 | 1.003-1.058 | 0.027 |
| Gender | 4.848 | 0.262 | 1.300 | 1.029-1.641 | 0.028 |
| Creatinine | 4.063 | 0.005 | 1.005 | 1.000-1.011 | 0.044 |
| Hypertension | 3.039 | 0.167 | 1.182 | 0.979-1.427 | 0.081 |
| T2DM | 1.353 | -0.137 | 0.872 | 0.692-1.099 | 0.245 |
| Smoking history | 1.250 | 0.114 | 1.120 | 0.918-1.368 | 0.264 |
| Anemia | 0.666 | 0.263 | 1.301 | 0.692-2.446 | 0.415 |
| NSTEMI | 0.491 | 0.077 | 1.080 | 0.870-1.341 | 0.484 |
| Family history of CAD | 0.064 | 0.035 | 1.056 | 1.031-1.081 | <0.001 |

*LVEF* left ventricular ejection fraction, *GA* glycated albumin, *LM* left main artery, *TC* total cholesterol, *SYNTAX* synergy between PCI with taxus and cardiac surgery, *BMI* body mass index, *T2DM* type 2 diabetes mellitus, *NSTEMI* non-ST-segment elevation myocardial infarction, *CAD* coronary artery disease

## Table S4. Parameters for the baseline + FBG model (sort by Chi square value in descending order)

|  | Chi-square value | β | HR | 95% CI | *P* value |
| --- | --- | --- | --- | --- | --- |
| LVEF | 37.300 | -0.037 | 0.964 | 0.952-0.975 | <0.001 |
| Age | 36.681 | 0.033 | 1.033 | 1.022-1.044 | <0.001 |
| Multi-vessel lesion | 22.328 | 0.644 | 1.904 | 1.458-2.486 | <0.001 |
| FBG | 16.343 | 0.079 | 1.082 | 1.041-1.124 | <0.001 |
| LM lesion | 12.866 | 0.587 | 1.799 | 1.305-2.479 | <0.001 |
| TC | 8.774 | 0.120 | 1.127 | 1.041-1.220 | 0.003 |
| SYNTAX score | 7.146 | 0.026 | 1.026 | 1.007-1.046 | 0.008 |
| Gender | 5.231 | 0.272 | 1.312 | 1.040-1.656 | 0.022 |
| Creatinine | 4.945 | 0.006 | 1.006 | 1.001-1.011 | 0.026 |
| BMI | 3.090 | 0.024 | 1.024 | 0.997-1.052 | 0.079 |
| Hypertension | 2.714 | 0.158 | 1.171 | 0.970-1.414 | 0.099 |
| Smoking history | 1.198 | 0.111 | 1.117 | 0.916-1.363 | 0.274 |
| NSTEMI | 0.572 | 0.083 | 1.087 | 0.876-1.349 | 0.449 |
| Anemia | 0.572 | 0.244 | 1.276 | 0.678-2.401 | 0.449 |
| T2DM | 0.070 | -0.028 | 0.972 | 0.788-1.199 | 0.791 |
| Family history of CAD | 0.043 | 0.029 | 1.030 | 0.782-1.356 | 0.835 |

*LVEF* left ventricular ejection fraction, *FBG* fasting blood glucose, *LM* left main artery, *TC* total cholesterol, *SYNTAX* synergy between PCI with taxus and cardiac surgery, *BMI* body mass index, *NSTEMI* non-ST-segment elevation myocardial infarction, *T2DM* type 2 diabetes mellitus, *CAD* coronary artery disease

## Table S5. Parameters for the baseline + HbA1c model (sort by Chi square value in descending order)

|  | Chi-square value | β | HR | 95% CI | *P* value |
| --- | --- | --- | --- | --- | --- |
| Age | 36.721 | 0.033 | 1.033 | 1.022-1.044 | <0.001 |
| LVEF | 34.028 | -0.036 | 0.965 | 0.954-0.977 | <0.001 |
| Multi-vessel lesion | 21.292 | 0.629 | 1.875 | 1.436-2.450 | <0.001 |
| HbA1c | 17.681 | 0.190 | 1.209 | 1.107-1.321 | <0.001 |
| LM lesion | 13.314 | 0.597 | 1.816 | 1.318-2.503 | <0.001 |
| TC | 9.130 | 0.121 | 1.129 | 1.043-1.221 | 0.003 |
| SYNTAX score | 6.189 | 0.024 | 1.025 | 1.005-1.044 | 0.013 |
| Creatinine | 4.419 | 0.006 | 1.006 | 1.000-1.011 | 0.036 |
| Gender | 4.267 | 0.246 | 1.279 | 1.013-1.615 | 0.039 |
| Hypertension | 3.004 | 0.166 | 1.181 | 0.978-1.426 | 0.083 |
| BMI | 2.882 | 0.023 | 1.023 | 0.996-1.051 | 0.090 |
| T2DM | 2.051 | -0.185 | 0.831 | 0.645-1.071 | 0.152 |
| Anemia | 0.884 | 0.303 | 1.354 | 0.720-2.547 | 0.347 |
| Smoking history | 0.742 | 0.088 | 1.092 | 0.894-1.333 | 0.389 |
| NSTEMI | 0.408 | 0.071 | 1.073 | 0.864-1.332 | 0.523 |
| Family history of CAD | 0.009 | 0.014 | 1.014 | 0.770-1.335 | 0.922 |

*LVEF* left ventricular ejection fraction, *HbA1c* glycosylated hemoglobin A1c, *LM* left main artery, *TC* total cholesterol, *SYNTAX* synergy between PCI with taxus and cardiac surgery, *BMI* body mass index, *T2DM* type 2 diabetes mellitus, *NSTEMI* non-ST-segment elevation myocardial infarction, *CAD* coronary artery disease
